# Villoglandular adenocarcinoma of the uterine cervix: a systematic review and meta-analysis

**DOI:** 10.1007/s00404-021-06077-9

**Published:** 2021-05-25

**Authors:** Anna K. Dietl, Matthias W. Beckmann, Konrad Aumann

**Affiliations:** 1 Department of Obstetrics and Gynecology, University of Erlangen, 91054 Erlangen, Germany; 2 Center for Pathology Allgaeu, Klinikverbund Allgaeu, Kempten, Germany; 3 Faculty of Medicine, University of Freiburg, 79106 Freiburg, Germany

**Keywords:** Villoglandular adenocarcinoma, Cervix, Conservative therapy, Invasive therapy, Review

## Abstract

**Purpose:**

Villoglandular adenocarcinoma (VGA) of the uterine cervix has been classified as a rare subtype of cervical adenocarcinoma with good prognosis. A conservative surgical approach is considered feasible. The main risk factor is the presence of other histologic types of cancer.

In this largest systematic review to date, we assess oncological outcomes associated with conservative therapy compared to those associated with invasive management in the treatment of stage Ia and Ib_1_ VGA.

**Methods:**

Case series and case reports identified by searching the PubMed database were eligible for inclusion in this review (stage Ia–Ib_1_).

**Results:**

A total of 271 patients were included in our literature review. 54 (20%) patients were treated by “conservative management” (conization, simple hysterectomy, and trachelectomy) and 217 (80%) by “invasive management” (radical hysterectomy ± radiation, hysterectomy, and radiation). Recurrences of disease (RODs) were found in the conservative group in two (4%) cases and in the invasive group in nine (4%) cases. There was no significant difference in disease-free survival (DFS) according to conservative or invasive treatment (*p *= 0.75). The histology of VGA may be complex with underlying usual adenocarcinoma (UAC) combined with VGA.

**Conclusion:**

The excellent prognosis of pure VGA and the young age of the patients may justify the management of this tumor using a less radical procedure. The histological diagnosis of VGA is a challenge, and pretreatment should not be based solely on a simple punch biopsy but rather a conization with wide tumor-free margins.

## Introduction

Adenocarcinoma of the cervix comprises for 15–20% of all carcinomas of the uterine cervix. There is evidence that the absolute incidence of adenocarcinoma is increasing, especially in women younger than 35 years [1, 2].

In 1989, Young and Scully [3] drew attention to a rare subtype of cervical adenocarcinoma, the villoglandular adenocarcinoma (VGA). The International Endocervical Adenocarcinoma Criteria and Classification (IECC) declared that VGA is a human papillomavirus (HPV)—associated adenocarcinoma [4]. The incidence of this subtype is reported as 4–9% of usual cervical adenocarcinoma (UAC) [5, 6].

The standard surgery for patients with stage Ia_2_–Ib_1_ cervical cancer is radical hysterectomy (RH) and lymphadenectomy (LNE). However, this procedure does not preserve fertility and can significantly affect quality of life.

The majority of reports revealed that the long-term prognosis of VGA is more favorable than UAC. Non-radical surgery or ovarian preservation might be safe for patients with pure early-stage VGA.

The aim, on the one hand, should be to avoid overtreatment by determining an exact diagnosis to preserve the fertility of young women and, on the other hand, to identify risk factors and offer optimal therapy for the VGA-tumor. Thus, the choice of treatment in patients with VGA remains controversial, and clarity is needed. In this largest systematic review to date, we assess oncological outcomes associated with conservative therapy compared to those associated with invasive management in the treatment of stage Ia and Ib_1_ VGA.

## Materials and methods

**Fig. 1:**
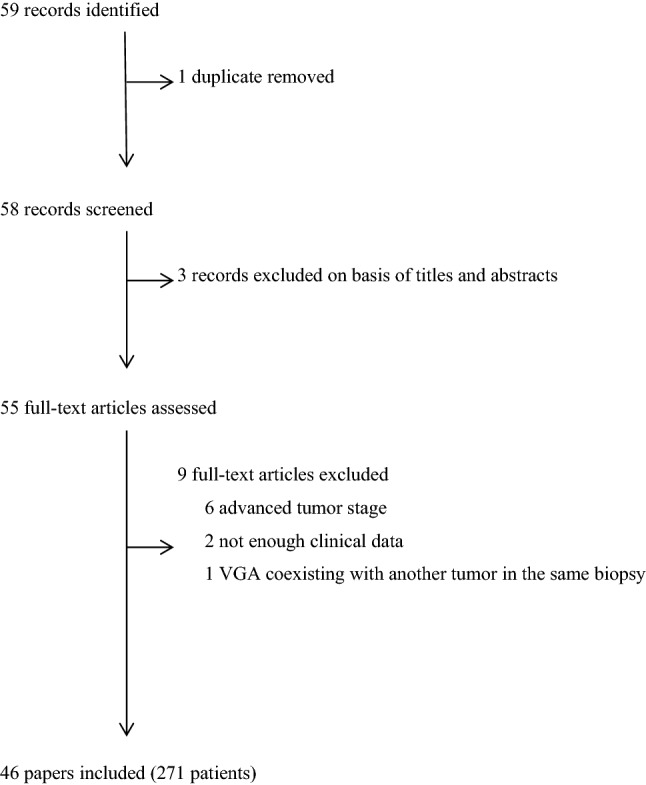
Search strategy and exclusion criteria (adapted from PRISMA [7])

This systematic review was based on the PRISMA guidelines [7] (Fig. 1). Published reports were identified by searches of PubMed and from references of relevant articles published from 1989 (the year VGA was described by Young and Scully [3]) to 2021. We used the search terms “villoglandular adenocarcinoma of the uterine cervix”, “early-stage cervical cancer”, “cone biopsy”, and “radical hysterectomy”. All papers that reported VGA in the abstracts and contained adequate information (including patient age, stage, primary treatment and postoperative treatment (radiotherapy and chemotherapy), clinical course, and follow-up) were included. The review included only women with “early-stage cervical cancer” and excluded patients with stages Ib_2_–IIIb. Tumors were included if they were in stage I not otherwise specified (nos) when a study was published before the subdivision into Ib_1_ and Ib_2_ was implemented and if the tumor would be classified as stage Ib_1_ according to a relevant clinical and pathological description.

If the preoperative diagnosis was made only by a single biopsy (punch biopsy), then the final diagnosis with the surgical specimen (cervix, uterus) did not always confirm the initial diagnosis due to the fact that small biopsies often contain tissue from the surface of the exophytic tumor only. For a proper diagnosis of VGA histological evaluation of tumor including its basis is mandatory. In 14 cases, the diagnosis of VGA was made primarily by single biopsy, and the surgical specimen resulted in *n* = 5 VGA + UAC, *n* = 3 VGA + squamous cell carcinoma, *n* = 5 UAC, and *n* = 1 endometrial adenocarcinoma [8–17]. These cases were excluded from the study.

To compare disease-free survival (DFS) distributions between conservative and invasive treatment groups, a meta-analysis including a total of 44 papers and a total of 232 patients was carried out. Whenever individual follow-up data were not available, they were estimated by equidistantly dividing the respective time intervals. Statistics were calculated using SPSS Version 25. Data analysis was performed with descriptive statistics and Kaplan–Meier curves. DFS outcomes were compared with the log rank test.

## Results

**Fig. 2:**
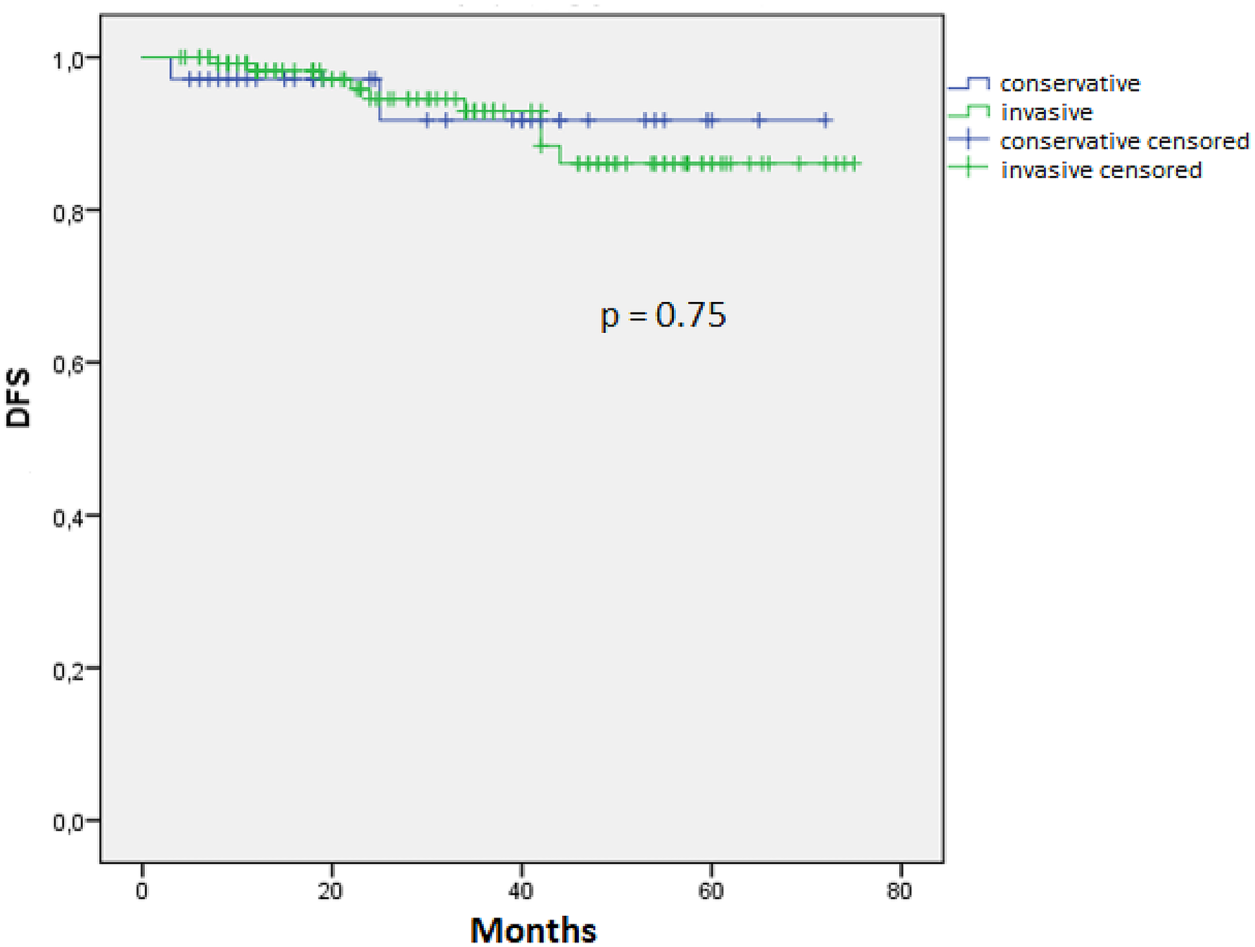
DFS of VGA by conservative and invasive treatment

The PubMed search generated 59 reports and comprised a total of 398 patients. Of these, 271 patients met the inclusion criteria and underwent conservative management (*n* = 54: conization, simple hysterectomy, trachelectomy, without adjuvant therapy) or invasive management (*n* = 217: radical hysterectomy (RH) with or without adjuvant therapy, hysterectomy with adjuvant therapy). There was no significant difference in DFS according to conservative or invasive treatment (Fig. 2, log rank, *p* = 0.75).

### Conservative management

We found 21 reports (stage Ia_1_, Ib_1_, I nos) describing conization in 28 patients, hysterectomy in 21 patients, and trachelectomy in five patients. Nine patients underwent pelvic lymph node dissection, lymph node biopsy or lymph node sampling (Table 1).

**Table 1:** Literature review of conservative management for VGA

	Number of patients	Average age, years (range)	FIGO stage	Surgery	Outcome (follow-up, months)
Young and Scully 1989 [3]	6	33 (23–54)^a^	I nos	1 CON	NED (24–168)^a^
				5 SH	
Jones et al. 1993 [32]	5	37 (27–54)^a^	I nos	CON	NED (13–55)
Skopelitou and Hadjiyannakis 1996 [44]	1	21	Ib_1_	CON	NED (12)
Novotny and Ferlisi 1997 [45]	3	35 (25–48)	I nos	2 CON	NED (9–32)
				1 SH	
Borgo et al. 1998 [46]	1	26	Ib_1_	CON	NED (40)
Bouman et al. 1999 [9]	1	26	Ib_1_	CON	NED (15) delivery 15 months after CON at 36 weeks
Chang et al. 1999[47]	2	40 (35–44)	I nos	SH	NED (8–11)
Hoffman et al. 2001 [20]	1	28	Ib_1_	CON (amputation of the cervical portio)	NED (40) delivery at 36 weeks
Falcon et al. 2006 [21]	1	34	Ib_1_	CON	NED (96) delivery 60 months after CON
Macdonald et al. 2006 [8]	1	32	Ib_1_	CON	ROD 3 months after CON recurrence (cervix), underwent RAD/CT, DOD (tumor progression, UAC, second opinion)
Lavie et al. 2008[18]	1	31	Ib_1_	CON (14th week of gestation)	NED (18)
				CRH (37th week)	
Korach et al. 2009[10]	5	42 (33–65)	2 Ib_1_ 3 Ia_1_	2 CON	NED (72–120) 1 term delivery
				2 SH	
				1 SH + BSO + LN sampling	
Takai et al. 2010 [19]	1	28	Ib_1_	CON (16 weeks of gestation)	NED (44) delivery at 38 weeks
Hagiwara et al. 2013 [28]	1	34	Ib_1_	SH + LN-biopsy	NED (154)
Lataifeh et al. 2013 [22]	3	30 (27–32)	Ib_1_	1 CS and CON, trachelectomy and LNE	NED (6–60)
				2 trachelectomy and LNE	
Kim et al. 2014 [5]	5	37 (32–44)	3 Ia_1_ 2 Ib_1_	4 CON	4 NED (18–55) 1 ROD 25 months after CON recurrence (cervix), underwent RH, NED (62)
				1 LAVH + LNE (Ia_1_)	
Dilley et al. 2015 [48]	2	35 (33–37)	Ib_1_	1 CON	NED (18–41)
				1 RoHE	
Guo et al. 2018 [6]	3	32 (28–35)	Ib_1_	2 CON	NED (5–19)
				1 vag. trachelectomy + LNE	
Ju et al. 2018 [24]	3	43 (28–56)	1 Ib_1_ 2 Ia_1_	1 CON	NED (44–65)
				1 VH + BSO	
				1 TLH	
Wei et al. 2018 [17]	4	37 (24–55)	Ib_1_	2 CON	NED (22–38)
				1 TLH + BSO	
				1 TLH + BSO + LNE	
Chen et al 2021 [29]	4	45 (38–52)	3 Ib_1_ 1 Ia_2_	1 SH + BS	NED (25–90)
				1 SH + BSO	
				1 TLH + BS	
				1 trachelectomy + LNE	

*CON*  conization, *CS* cesarean section, *CRH* cesarean radical hysterectomy, *LAVH* laparoscopic-assisted vaginal hysterectomy, *SH*  simple hysterectomy, *VH*  vaginal hysterectomy, *TLH* total laparoscopic hysterectomy, *RoHE* robot-assisted hysterectomy, *BSO* bilateral salpingo-oophorectomy, *BS* bilateral salpingectomy, *RAD*  radiation, *CT*   chemotherapy, *nos*  not otherwise specified, *UAC*  usual adenocarcinoma. *LNE*  lymphadenectomy, *DOD*  dead of disease, *NED*  no evidence of disease, *ROD* recurrence of disease

^a^Including all patients of both groups

Negative LVI (lymphovascular invasion) status was reported in 32 patients, positive LVI status was reported in no patients, and LVI status was not reported in 22 patients.

Two patients (4%) had recurrent disease: one in the cervix 25 months after conization [5]. The reported margins of the conization were uninvolved but were close to the tumor. She underwent RH and was alive after 62 months of follow-up. The second patient had a cone biopsy (VGA-tumor, 2.4 mm depth invasion, all resection margins clear) [8]. Close follow-up was recommended due to the histology. 3 months later, a cervical recurrence was noted. A biopsy showed a continuum from a well-differentiated adenocarcinoma with a villoglandular pattern to a poorly differentiated carcinoma. Rapid tumor progression followed chemoradiation therapy, and the patient died due to complications of an extensive pelvic tumor. Histology was sent for external review and was classified as a well-differentiated adenocarcinoma with a marked villoglandular pattern.

Seven pregnancies were reported in the “conservative management” group. In two patients, successful pregnancies were achieved following conization at the 14th/16th week of gestation [18, 19]. Four patients delivered 1–5 years after the conization [9, 10, 20, 21]. One patient received a punch biopsy and conization during pregnancy and later underwent a trachelectomy and lymphadenectomy (LNE) of the tumor during cesarean section [22].

### Invasive management

We found reports of 217 patients with tumor stages Ia_1_, Ia_2_, Ib_1_, and I nos (Table 2). Recurrent disease was seen in nine (5%) patients, and three deaths were reported.

**Table 2:** Literature review of invasive management for VGA

	Number of patients	Average Age, year (range)	FIGO stage	Surgery and/or additional treatment	Outcome (follow-up, months)
Young and Scully 1989 [3]	7	33 (23–54)^a^	I nos	4 RH + LNE	NED (24–168)^a^
				3 RH	
Hopson et al. 1990 [13]	3	36 (28–42)	Ib	3 RH + LNE	NED (1 uneventful hospital course, 2: 8mths)
Jones et al. 1993 [32]	19	37 (27–54)^a^	I nos	4 SH + RAD	NED (7–77)^a^
				15 RH	
Reed et al. 1993 [49]	4	34 (25–43)	Ib	1 SH + CT	NED (18–28)
				3 RH + LNE + CT	
Hurteau et al. 1995 [30]	1	22	Ib	CRH + LNE 32 weeks gestation	NED (14)
Kaku et al. 1997 [12]	5	45 (33–54)	Ib	5 RH + LNE + BSO (1 LN +) + 1 RAD	NED (9–169)
Stanley-Christian et al. 1997 [14]	3	34 (27–41)	Ib_1_	RH + LNE + BSO	NED (publication date)
Lu et al. 1998 [50]	1	47	Ib_1_	RH + LNE	NED (9)
Bouman et al. 1999 [9]	2	34 (29–38)	Ib	1 RH + LNE	NED (recovery uneventful)
				1 SH + RAD	
Chang et al. 1999 [47]	1	42	Ib	SH + RAD	NED (13)
Lakhtakia et al. 2000 [51]	1	30	Ib	RH + LNE + CT	NED (9)
Lellé et al. 2000 [52]	1	45	I nos	RH + LNE	NED (9)
Khunamornpong et al. 2001 [11]	14	38 (22–49)	Ib	12 RH + LNE	NED (21–144)
				2 RH + LNE + RAD (2 LN +)	
Reale et al. 2001 [53]	1	69	I nos	RH + LNE	NED (60)
Polat et al. 2002 [54]	1	38	I nos	RH + LNE	NED (28)
Garcea et al. 2003 [27]	1	29	Ib_1_	RH + LNE + RAD (LN +)	NED (34)
Dede et al. 2004 [26]	1	28	Ib_1_	After termination of the pregnancy at 8 weeks RH	ROD (42), DOD (“on the fifth year of first diagnosis”)
Utsugi et al. 2004 [55]	10	45 (36–64)	Ib_1_	9 RH + LNE	NED (36–228)
				1 RH + LNE + CT	
Fadare and Zheng 2005 [16]	1	47	Ib_1_	RH + LNE + BSO	NED (4,5)
Heron et al. 2005 [23]	1	32	Ib_1_	Delivery 38 weeks, VGA (cervical polyp), 1 month pp: RH + LNE	ROD (44) (episiotomy scar)
					NED (96)
Gonzalez-Bosquet et al. 2009 [56]	1	28	Ib	RH + LNE	NED (18)
Korach et al. 2009 [10]	3	39 (34–65)	Ib_1_	3 RH + LNE + BSO	2 NED (78–180)
					1 ROD (24), DOD (“few months later”)
Lai et al. 2011 [25]	12	42 (32–52)	10 Ib_1_ 2 Ia_2_	9 RH + LNE + BSO	11 NED (34–162)
				2 RH + LNE (1 LN +)	1 ROD (alive 153 mths)
				1 LNE + RAD/CT	
Choi et al. 2012 [57]	2	52 (48–55)	Ib_1_	1 RH	NED (13–23)
				1 RH + LNE + BSO	
Hagiwara et al. 2013 [28]	5	37 (30–41)	Ib_1_	4 RH + LNE ± BSO	NED (42–128)
				1 RH + LNE + RAD (1 LN +)	
He 2013[31]	1	31	Ib_1_	Biopsy at 28 weeks (cervical papilloma), CRH + LNE (36 weeks)	NED (84)
Lataifeh et al. 2013 [22]	8	37 (29–49)	Ib_1_	6 RH + LNE + Brachy	NED (18–120)
				1 RH + LNE + RAD/CT (1 LN +)	
				1 RH + LNE	
Kim et al. 2014 [5]	8	47 (34–72)	Ib_1_	2 RH + LNE	NED (9–150)
				1 RH + LNE + BSO + RAD	
				2 RH + LNE + USO	
				1 LRH + LNE + USO + RAD	
				1 RH + LNE + BSO	
				1 VH + RAD	
Takeuchi et al. 2014 [58]	1	38	I nos	RH	NED (publication date)
Zhao et al. 2016 [15]	6	36 (31–42)	Ib_1_	2 RVH + LNE + BSO + AT	NED (7–57)
				2 RVH + LNE + USO + AT	
				2 RVH + LNE + BSO	
Zhou et al. 2016 [59]	4	55 (47–70)	Ib_1_	2 RH + LNE + BSO	NED (49–83)
				1 RH + LNE	
				1 SH + RAD	
Niu et al. 2017 [60]	4	55 (47–70)	Ib_1_	1 SH + RAD	3 NED (8–34)
				1 amputation of cervix + LNE	
				1 RH + LNE	1 NED (publication date)
				1 RH + LNE + BSO	
Guo et al. 2018 [6]	32	42 (27–66)	3 Ia_1_ 1 Ia_2_ 28 Ib_1_	12 RH + LNE + BSO	31 NED (6–104)
				8 LRH + LNE + BSO	
				4 NSLRH + LNE + BSO	
				1 LRH + BSO	1 ROD (8), (pelvic, adenocarcinoma), AWD (37)
				3 LRH + LNE + BS	
				1 RVT + LNE	
				1 NSARH + LNE + BS	
				1 LRH + BS	
				1 CS + RH + LNE + BS Including 9 patients with neo-/adjuvant treatment (CT and/or RAD)	
Ju et al. 2018 [24]	11	49 (31–64)	10 Ib_1_ ﻿1 Ia_2_	3 MRH + LNE + BSO	7 NED
				2 LRH + LNE + BSO	1 ROD (22)(vaginal stump)
				2 LMRH + BSO	1 ROD (42) (liver)
				1 RH + LNE + BSO + RAD/CT (1 LN +)	1 ROD (34) (adnexa)
				1 RH + LNE + BSO	1 ROD (12) (adnexa), DOD (42)
				1 LRH + LNE	
				1 LMRH	
Wei et al. 2018 [17]	6	42 (31–50)	Ib_1_	RH	NED (5–113)
				RH + LNE	
				RH + BSO + LNE	
				LRH + LNE	
				LRH + BSO + LNE	
				LRH + BSO + LNE + CT/RAD	
Zhang et al. 2020 [61]	3	46 (37–58)	Ib_1_	2 RH + LNE + BSO	NED (56–120)
				1 RH + LNE + BSO + CT	
Chen et al. 2021 [29]	35	43 (32–68)	32 Ib_1_ 3 Ia_2_	19 RH + LNE + BSO (1 LN +)	NED (5–152)
				10 LRH + LNE + BSO (1 LN +)	
				3 RH + LNE + BS	
				1 LRH + LNE + BS	
				1 SH + LNE + BS + RAD/CT	
				1 SH + BS + CT Including further 11 patients with adjuvant treatment (CT and/or RAD)	

*USO* unilateral salpingo-oophorectomy, *VH* vaginal hysterectomy, *SH* simple hysterectomy, *LRH* laparoscopic radical hysterectomy, *RVT*  radical vaginal trachelectomy, *RVH*  radical vaginal hysterectomy, *AT*  adjuvant treatment, *LRH* laparoscopic radical hysterectomy, *NSLRH*  nerve-sparing laparoscopic radical hysterectomy, *BS* bilateral salpingectomy, *LMRH*   laparoscopic modified radical hysterectomy, *CRH*  cesarean radical hysterectomy, *RAD * radiation. *CT*  chemotherapy, *pp*  post-partum. *Brachy*  brachytherapy, *LN*   lymph node, *nos* not otherwise specified, *AWD*  alive with disease, *NED* no evidence of disease, *ROD* recurrence of disease, *DOD*  dead of disease

^a^Including all patients of both groups

Among the nine patients with recurrence, one patient with FIGO stage Ib_1_ received a nerve-sparing laparoscopic RH, bilateral salpingo-oophorectomy, and pelvic LNE [6]. The histology showed well-differentiated VGA, and the infiltration depth was 5 mm. The tumor recurred in the pelvic cavity after 8 months. At explorative laparotomy, the pelvic tumor was removed, and the histology revealed a UAC.

Another patient showed a VGA of the cervix after an uncomplicated vaginal delivery, and an RH with LNE was performed [23]. 44 months thereafter, the VGA recurred in the episiotomy scar.

One patient in the study by Korach et al. [10] was initially misdiagnosed with VGA instead of cervical adenocarcinoma. The tumor recurred 2 years after RH, and the patient died a few months later.

The case series of Ju et al. [24] reported four metastases after RH, two in the ovaries, one in the liver and one on the vaginal vault. One patient had progressive disease after bilateral salpingo-oophorectomy because of ovarian metastasis and died 30 months later. The three patients with intraabdominal metastasis all underwent laparoscopic RH.

The case series of Lai et al. [25] reported one case of recurrence after RH, bilateral salpingo-oophorectomy, and LNE. The patient was relapse-free for 153 months.

Dede et al. [26] reported a patient at 8 weeks of gestation who received a cervical punch biopsy revealing a VGA. After termination of the pregnancy, RH was performed. The tumor recurred in the pelvis 42 months after primary surgery. The patient died because of tumoral complications 5 years after the diagnosis of the disease.

In nine patients, at least one affected lymph node could be detected [5, 11, 12, 22, 25, 27–29], three showed a positive LVI [5, 12, 28], and three was LVI negative [27, 29]. In two patients, LVI was not reported [22, 25].

Four children were born in the “invasive management” group: three by cesarean section combined with RH [6, 30, 31] and one spontaneously [23].

## Discussion

Stage Ib_1_ cervical cancer is typically treated with invasive management (RH or primary chemoradiation). Several histologic subtypes have been defined, and the particular subtypes may affect prognosis and thus treatment decisions.

VGA has been described as a separate subtype of adenocarcinoma of the cervix; it is well-differentiated and usually associated with a favorable outcome [3, 32]. The preoperative selection of young patients is an important issue because of the possibility for fertility-sparing or less-invasive treatment. In the “conservative management” group, 54 patients were treated with conization, simple hysterectomy or trachelectomy without adjuvant therapy (radiation, chemotherapy). One patient had recurrent disease in the cervix 25 months after conization [5]. The margins of excision were uninvolved but were close to the tumor. Analysis of adenocarcinoma in situ indicates that achieving negative margins after surgical excision is associated with a significantly lower rate of residual or recurrent disease [33]. The risk of recurrence was lower for patients who underwent a secondary excisional procedure. Goldstein and Mani [34] reported that the risk of residual disease was reduced when a disease-free margin of 10 mm was achieved.

VGA is frequently associated with adenocarcinoma in situ (40%) or cervical intraepithelial neoplasia (30%) [32]. The selection of appropriate patients for “conservative management” has been hampered by uncertainty regarding the natural history of VGA and associated risk of recurrence along with the potential for multifocal lesions that extend beyond the margin of an otherwise satisfactory conization. To maintain fertility in young patients, a conization with a wide disease-free margin, possibly by performing a second resection, should be the goal.

Other histological factors that should be taken into account are depth of stromal invasion and LVI status. These are prognostic factors for recurrence in early-stage cervical cancer [35] and cannot reliably be assessed in a biopsy specimen alone. Grossly, VGA tumors present as friable or polypoid masses, usually protruding from the endocervical canal and manifesting macroscopically as Ib tumors but often with only superficial or no stromal invasion, similar to Ia tumors. To this end, histological evaluation of the tumor-stroma border is necessary. However, approximately 80% of VGA tumors are radically treated and thus are very often overtreated. Over 95% of stage I VGA tumors have no or only superficial stromal invasion, and only 3% are LVI positive [6].

In the present review, one positive lymph node was described in the invasive group in nine patients [11, 12, 22, 24, 25, 27–29], whereby four patients were LVI positive, one was negative; in two cases, LVI had not been determined. Six patients were irradiated postoperatively, and no recurrence occurred. Since lymph node involvement was detected in individual cases with VGA, LNE, e.g., laparoscopic pelvic LNE, remains an option (at least in LVI positive patients) even in the case of uterus preservation.

The patient who died in the “conservative management” group had a VGA diagnosed via conization. However, an external review revealed a VGA with an underlying well-differentiated adenocarcinoma [8]. Alfsen et al. [36], in studying the reproducibility of histological classification of nonsquamous-cell carcinomas of the uterine cervix, reported agreement between reviewers in only 3 of 15 cases of VGA. The nature of accurate histologic diagnosis of VGA is challenging because of the high rate of pretreatment misdiagnosis [8–10, 37]. A punch biopsy prior to treatment very often yielded an incorrect histological diagnosis. Obviously, it can be difficult to predict the final histopathology via examination of a single biopsy, even if poor prognostic features are not present and the VGA seems to be the only entity [9]. Before definitive conservative management is considered, it is prudent to perform conization to exclude the presence of concomitant tumors and to definitively render the diagnosis of VGA. Moreover, in difficult borderline cases consultation of a second pathologist may be necessary.

In addition to the sometimes difficult histological diagnosis of pure VGA, the question of cell spillage due to manipulation of the exophytically growing primary tumor at the cervix is an additional problem [38]. If VGA is present at the cervix at the time of termination of pregnancy or during childbirth, the probability of tumor dissemination is very high. Tumor disseminations at birth are the main concerns for vaginal delivery through a cervix with cancer [39]. This explains the recurrences in this review [23, 26]. If the VGA had been removed via conization before the termination of pregnancy or before birth, a relapse would most likely not have occurred.

The three cases of intraabdominal metastases after minimally invasive surgery in the paper by Ju et al.[24] are probably also related to this problem. These three patients had no risk factors for metastasis (no LVI and no lymph node involvement and had superficial invasion only). In all 15 cases, VGA was diagnosed after a punch biopsy. Among potential reasons for the inferior oncological outcomes in patients with cervical cancer who underwent minimally invasive surgery than in women who underwent open surgery, the routine use of a uterine manipulator might increase the propensity for tumor spillage intraperitoneally after colpotomy under laparoscopic vision [40, 41].

The present literature review provides some evidence that the manipulation (“excessive handling”) of cervical VGA can worsen the prognosis of this tumor. Of the 11 cases of recurrence, the vast majority could most likely have been avoided if, first, the VGA at the cervix had been preventively removed by conization with tumor-free margins and, second, the exact histological diagnosis had been made by a qualified gynecopathologist.

The strengths of this study include the largest systematic review 1989–2021 of this rare tumor and the first attempt to compare a non-radical (conservative) with a radical (invasive) approach. However, our conclusions were limited by the retrospective view of the data and the number of VGA tumors was limited for this rare tumor. Thus, it is suggested to perform multicenter prospective studies to investigate diagnosis and optimal treatment of this subtype of cervical cancer.

The DFS of the conservative group is comparable to the invasive group (*p* = 0.75). Radical surgery in the invasive group does not lead to better results compared to the conservative group. Since these VGA tumors can always be visualized on gynecologic examination due to their exophytic growth and are accordingly classified as stage Ib (FIGO), most patients in the invasive group were treated with radical hysterectomy, as standard therapy for cervical cancer, although conization with wide negative margins would most likely have been sufficient for diagnosis and therapy. It would still have been possible to modify the therapy after conization depending on the stromal infiltration in the sense of a “patient-tailored surgical treatment”. In addition, conization can improve the prognosis of common cervical carcinoma [42].

Histopathological evidence of VGA should be included in the treatment decision and prognosis estimation in the multidisciplinary tumor conference. It is essential that VGA is treated as a special subtype of cervical carcinoma with an excellent prognosis. Awareness of this special form and decision-making strictly based on the histology of the conisate regarding possible further conservative or invasive therapy should be present.

In conclusion, VGA is a complex tumor that has an excellent prognosis in its pure histological appearance. It is not justified to lump VGA and usual cervical cancer together and to perform radical surgery. In any case, the decisive step towards adequate treatment for VGA is a qualified histological diagnosis that excludes a less differentiated carcinoma component. A pretherapeutic conization with wide tumor-free margins is an indispensable prerequisite for this decision. We believe that patients could benefit from this low-risk histology and the next step could be only a sentinel node mapping [43]. In pure VGA, conservative management is justifiable, especially for young women, and a radical approach may result in overtreatment.

## Declarations

### Availability of data and material

The datasets used and/or analyzed during for the presented manuscript are available from the corresponding author on reasonable request.
